# Contrastive Learning Pre-Training and Quantum Theory for Cross-Lingual Aspect-Based Sentiment Analysis

**DOI:** 10.3390/e27070713

**Published:** 2025-07-01

**Authors:** Xun Li, Kun Zhang

**Affiliations:** School of Computer Science and Engineering, Nanjing University of Science and Technology, Nanjing 210094, China; 219106010015@njust.edu.cn

**Keywords:** natural language processing, deep learning, cross-lingual ABSA, contrastive learning, quantum theory, quantum projection, quantum entanglement

## Abstract

The cross-lingual aspect-based sentiment analysis (ABSA) task continues to pose a significant challenge, as it involves training a classifier on high-resource source languages and then applying it to classify texts in low-resource target languages, thereby bridging linguistic gaps while preserving accuracy. Most existing methods achieve exceptional performance by relying on multilingual pre-trained language models (mPLM) and translation systems to transfer knowledge across languages. However, little attention has been paid to factors beyond semantic similarity, which ultimately hinders classification performance in target languages. To address this challenge, we propose CLQT, a novel framework that combines contrastive learning pre-training with quantum theory to address the cross-lingual ABSA task. Firstly, we develop a contrastive learning strategy to align data between the source and target languages. Subsequently, we incorporate a quantum network that employs quantum projection and quantum entanglement to facilitate effective knowledge transfer across languages. Extensive experiments reveal that the novel CLQT framework both achieves strong results and has a beneficial overall influence on the cross-lingual ABSA task.

## 1. Introduction

**Figure 1:**
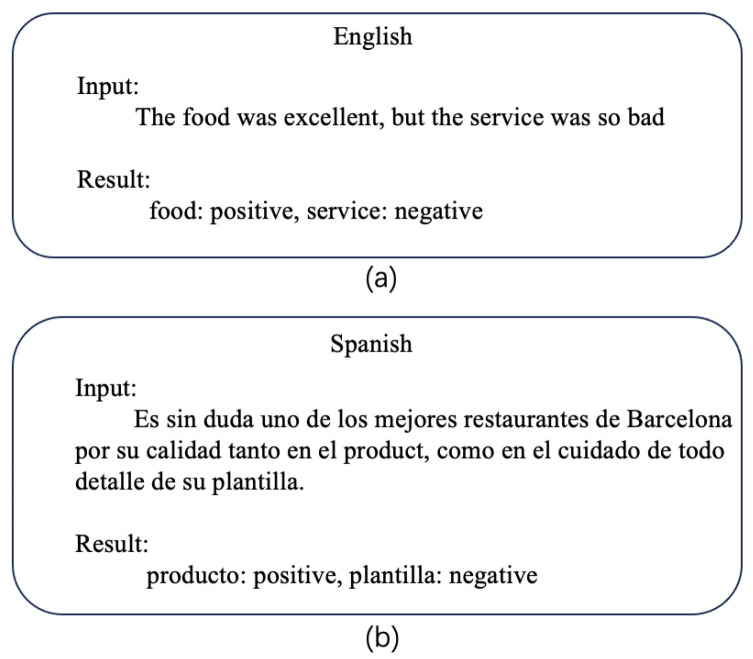
An example of cross-lingual aspect-based sentiment analysis. (**a**) is an English sample and (**b**) is a Spanish sample.

Aspect-based sentiment analysis (ABSA) is defined as extracting aspect information from text and determining the corresponding sentiment polarity [1,2,3]. For example, in a restaurant review, “The food is great, but the service was awful” in Figure 1. In this sentence, there are two aspect terms, “food” and “service,” respectively. The polarity of two aspects is food (positive) and service (negative). The vast availability of public opinion data allows stakeholders to formulate strategic plans by analyzing the ratio of favorable feedback. However, manually examining and interpreting a large number of user comments is both labor-intensive and costly, emphasizing the necessity for an automated approach.

Cross-lingual sentiment analysis (CLSA) involves training a classifier using source languages, typically resource-rich languages, and applying it to the target languages [4,5,6], as illustrated in Figure 1b. In contrast to monolingual sentiment analysis, CLSA accounts for structural and meaning variations across different linguistic systems, facilitates cross-linguistic knowledge transfer, and enables sentiment analysis models trained on data-rich languages to be effectively adapted for studying low-resource languages. This approach effectively tackles the challenge of limited sentiment resources in many non-English languages.

In recent years, multilingual BERT(mBERT) models and XLM-Roberta (XLM-R) models have become essential tools for addressing cross-lingual aspect-based sentiment analysis (ABSA) [7,8]. These pre-trained models acquire multilingual knowledge during their initial training phase and are subsequently adapted using annotated data from a high-resource language (typically English), allowing them to be applied to other target languages [9]. To further strengthen the transfer of language-specific information for cross-lingual aspect-based sentiment analysis, Zhang et al. [10] proposed a method that incorporates lexical alternation alongside a knowledge compression technique, aiming to enhance the alignment of these pre-trained models across different linguistic systems.

Despite substantial advancements in multilingual pre-trained models, their effectiveness in ABSA across different languages remains challenging, mainly due to the inadequate representation of low-resource languages during the pre-training process. One usual method for transferring language-specific knowledge is to use translated target language data with corresponding labels [11]. However, the effectiveness of methods relying on translated text is heavily dependent on translation accuracy and precise label alignment. Therefore, obtaining high-quality parallel translation data and eliminating translation errors remains a long-standing challenge.

Quantum mechanisms offer distinct advantages for cross-lingual ABSA [12]. In particular, quantum entanglement allows the model to encode correlations among various linguistic elements, such as aspect terms, opinion words, and sentiment polarities, simultaneously, thereby capturing intricate dependencies that traditional approaches might overlook. These entangled representations facilitate a more precise interpretation and analysis of sentiment information. Furthermore, quantum superposition enables a quantum system to exist in multiple states concurrently, a property that is particularly beneficial for NLP. Within a Hilbert space, superposition can represent several semantic layers at once, accommodating the inherent ambiguity and richness of language. Since words and phrases can convey different meanings depending on context, leveraging superposition allows the model to encode multiple semantic interpretations concurrently, thereby enhancing its understanding of complex linguistic structures. This capability renders the model more flexible and efficient in managing semantic variations and sentiment expressions across diverse languages.

**Figure 2:**
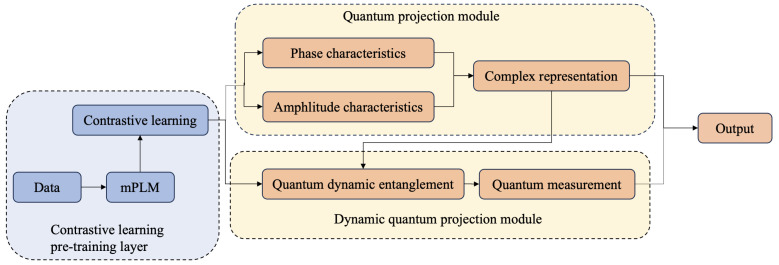
The CLQT framework is structured around three key components: contrastive learning, quantum projection, and a quantum entanglement module.

Zhao et al. [12] applied quantum entanglement to connect subsystems in quantum systems and resolving cross-lingual ABSA challenges through integration of quantum components within multilingual pre-trained models. However, this approach primarily relies on traditional multilingual models and does not effectively leverage the annotated data available in the high-resource domain. To address this limitation, we design a contrastive learning pre-training strategy that incorporates sentence translation to facilitate cross-lingual alignment and knowledge transfer. Contrastive learning is then employed to bring semantically similar sentences closer together while pushing semantically dissimilar sentences apart. This method not only maximizes the use of source language label knowledge but also reduces errors caused by translation quality. In contrast to [12], our method devises a dynamically adjustable entanglement state, which is governed by a linguistic similarity-weighted gating mechanism embedded in the quantum entanglement framework. The structural and grammatical differences between languages result in varying degrees of similarity across languages. For example, French and English are more similar than English and Russian. The quantum entanglement module proposed in [12] assumes equal influence across all languages, which is inconsistent with the nature of languages. To overcome this limitation, we introduce a dynamic entanglement module based on word vectors. Our proposed model, CLQT, combines contrastive learning pre-training with quantum mechanism networks, as shown in Figure 2.

We present CLQT, a novel framework combining quantum and contrastive learning pre-training, designed to enhance aspect sentiment analysis across multiple languages. This model consists of two main components: contrastive learning pre-training and quantum mechanism modules. The quantum mechanism module is further divided into the quantum projection module and the entanglement module. Specifically, to capture general knowledge across languages, the quantum projection module maps the text features obtained through contrastive learning pre-training into Hilbert space, where each aspect term is represented as a quantum superposition state. Additionally, to obtain language-specific knowledge, we design a quantum dynamic entanglement module, enabling the sharing of specific knowledge between languages through entangled representations. Finally, we connect the complex representations from both the quantum projection and entanglement modules to perform sequence labeling classification.

Our contributions are as follows:

1. This work proposes an innovative approach to cross-lingual ABSA that integrates contrastive learning pre-training with quantum mechanisms to enhance cross-lingual adaptability. To the best of our knowledge, this study is the earliest attempt to integrate these methodologies into aspect-based sentiment classification across multiple languages.
2. In the quantum mechanism module, we employ a dynamic gate based on language similarity to establish quantum entanglement states, which more effectively capture the varying degrees of similarity between languages.
3. We evaluated our CLQT model on the SemEval-2016 dataset [1]. The experimental results demonstrate the robustness of our proposed framework. Furthermore, a comprehensive evaluation was performed to analyze the contribution of each key module. The analysis further demonstrates the framework’s strong capability in transferring knowledge across languages.

## 2. Related Work

This section provides a concise overview of related research, encompassing aspect-based sentiment analysis, contrastive learning, and quantum networks.

### 2.1. Cross-Lingual Aspect-Based Sentiment Analysis

Conventional sentiment analysis is generally conducted at either the sentence or document level [13], while ABSA targets more fine-grained sentiment classification at the entity level [14]. The objective of cross-lingual ABSA is to develop an aspect-based sentiment classification model in one language and subsequently transfer it for use in another. Recent studies on cross-lingual ABSA have predominantly concentrated on subtasks involving the identification of aspect terms and the determination of their sentiment across different languages [15]. Most approaches utilize parallel corpora derived from machine translation, which help capture the semantic and syntactic knowledge of the target language [16]. Word or phrase alignment algorithms are then utilized to map label information between sentences in different languages [17], the effectiveness of these approaches largely relies on the accuracy of both translation and alignment. To enhance this approach, a technique leveraging multilingual word embeddings learned from extensive bilingual datasets has been introduced [18], allowing word embeddings to transfer across languages. Recently, transformer-based models pre-trained on extensive multilingual datasets, including multilingual BERT [7], XLM-Roberta [8], and mT5 [19], have achieved remarkable results across a range of cross-lingual NLP tasks. These models acquire multilingual knowledge during the pre-training phase from large corpora. These models are initially adapted using annotated examples in a high-resource language and are then directly utilized for predictions in the target language, exhibiting strong performance. To tackle the challenge of cross-lingual aspect-based sentiment classification, numerous studies have explored the integration of translation systems with pre-trained models, leveraging their ability to capture multilingual representations effectively [20,21]. To investigate the role of language-specific knowledge in cross-lingual aspect-based sentiment classification, Zhang et al. [10] proposed an aspect term code-switching (ACS) model.This architecture utilizes unsupervised learning mechanisms for aspect term extraction and is later adapted using annotated examples from a high-resource corpus, achieving results comparable to those of supervised approaches.

### 2.2. Contrastive Learning

The core idea behind contrastive learning is to create paired samples that enable the model to learn more distinct feature representations. Recently, this technique has achieved significant improvements across multiple natural language processing applications, mainly due to its ability to develop robust text embeddings [22]. Mitra et al. introduced a method combining memory networks and contrastive learning for cross-lingual stance detection [23]. Guo et al. applied contrastive learning to detect Alzheimer’s disease by bringing average vectors of confirmed cases closer together and pushing those of non-diagnosed cases further apart [24]. Li et al. proposed a representation learning strategy based on contrastive objectives to identify implicit sentiment in aspect-level sentiment classification [25]. Luo et al. enhanced the performance of contrastive learning by incorporating in-batch negative samples [26]. Zhou et al. integrated an emotion-aware thinking chain prompt module with contrastive learning to capture label relevance [27].

Contrastive learning has demonstrated its effectiveness across diverse natural language processing applications, particularly in learning semantic representations of labels within monolingual settings. However, traditional contrastive learning is constrained to within-language feature comparisons, often disregarding semantic relationships between different languages and sentences. Conventional methods primarily emphasize feature alignment within a single language, lacking the ability to fully leverage the rich cross-linguistic semantic information. This limitation becomes especially apparent in tasks that demand a deeper integration and comprehension of multiple semantic layers [28]. To address this issue, this paper strengthens representation learning by integrating sentence translations to create both similar and contrasting sample pairs, thereby enhancing overall performance.

### 2.3. Quantum Neural Network

Quantum neural networks (QNN) extend the functionality of traditional neural networks by leveraging quantum properties such as superposition and entanglement. Li et al. introduced an innovative fusion mechanism that incorporates quantum cognition into neural networks for emotion prediction. This method employs the quantum superposition state of judgments within a complex Hilbert space, utilizing positive operator-valued measures to classify samples as expressing either positive or negative emotions [29]. Yan et al. introduced an innovative quantum-based model for solving optimization challenges, employing a supervised learning framework to achieve enhanced performance [30]. Additionally, Zhou et al. proposed a generative adversarial network that integrates quantum and classical components to synthesize images by modeling discrete distributions [31]. Gong et al. construct a variational quantum circuit that incorporates quantum principles into convolutional neural networks to enhance model performance [32].

Building on recent progress in quantum-enhanced learning, we propose a novel framework for cross-lingual ABSA tasks. This framework combines quantum mechanisms with contrastive learning pre-training methods, aiming to improve performance in sentiment analysis across languages.

## 3. Method

This part presents the architecture of the CLQT model, outlining its key components and design principles. The model includes two main modules: contrastive learning pre-training strategy and quantum networks. In the pre-training phase, we apply a contrastive learning approach to reduce the semantic gap between source instances and their respective translated counterparts. Inspired by [12], in the fine-tuning phase, we employ quantum projection, quantum measurement, and quantum entanglement techniques to project both source and target languages into Hilbert space. We construct entangled states across multiple languages by the fundamental properties of quantum systems and using quantum measurements to compute the probability distributions for each aspect. Finally, we propose a parameterized rotation gate to replace the static Controlled-NOT (CNOT) gate, which enables dynamic adjustment of entanglement states based on the linguistic similarity between languages.

### 3.1. Problem Definition

Cross-linguistic sentiment analysis at the aspect level requires detailed text prediction and can be represented using a sequence labeling framework [33,34]. $x={\left\{x_{i}\right\}}_{i=l}^{N}$ represents a sentence, and the sentence length is *N*; d $x_{i}$ represents the *i*-th word; the model predicts the label sequence ${\left\{y_{i}\right\}}_{i=1}^{L}$, where $y_{i}\in Y=\left\{B,I,E,S\right\}-\left\{POS,NEU,NEG\right\}\cup \left\{O\right\}$ represents the label of word $x_{i}$, where *B* denotes the beginning of an aspect term, *I* represents the intermediate word, *E* denotes the end of the aspect term, and *S* represents a single aspect term. {POS,NEU,NEG} represent the polarity of the aspect term, and *O* represents non-aspect terms. For example, $y_{i}=B-NEG$ means the word $x_{i}$ is the beginning of a sentiment-negative aspect term. In this study, we only use the label from the source language $\left(x_{S},y_{S}\right)\in D_{S}$ to predict the $y_{T}$ for the target language text $x_{T}$.

### 3.2. Contrastive Learning Pre-Training

As a self-supervised approach, this technique aims to acquire meaningful data representations by analyzing similarities and distinctions among samples. Specifically, an anchor sample is used as a reference, positive samples are semantically similar to the anchor. In the training phase, the feature representations of positive samples are brought closer to the anchor. In contrast, negative samples, which are semantically dissimilar to the anchor, have their representations pushed further apart. In this study, we construct a contrastive learning module by representing source languages and their translated samples in a batch as anchor and positive samples, respectively, and all other samples within the batch are treated as negative instances. Contrastive learning pre-training strategy is described in Algorithm 1. Let $x=x_{i}^{N}$ denote a sentence, where *N* is the sentence length and $x_{i}$ represents the *i*-th word. We apply the multilingual pre-trained model (mPLM) to encode the sequence *x*, resulting in:

(1) $$H_{S}=\left\{h_{1},h_{2},\ldots ,h_{n}\right\}=mPLM\left(x_{1},x_{2},\ldots ,x_{n}\right)$$

$H_{T_{i}}$ represents the feature representation of the translated target language encoded through mPLM, where $T_{i}$ denotes the target language, *P* represents the total count of target languages, which we set to four in this study. $H_{N}$ represents the feature representation of non-translated samples. The pre-training contrastive learning loss function is defined as

(2) $$l_{cl}=\sum_{i=1}^{B}-log\frac{1}{P}\sum_{j=1}^{P}\frac{exp(Sim\left(H_{S_{i}},H_{T_{j}}\right)/\tau )}{\sum_{k=1,k\neq i}^{B}exp\left(Sim\left(H_{S_{i}},H_{N_{k}}\right)/\tau \right)}$$

Here *B* represents the number of samples in a batch; τ represents the temperature coefficient.
**Algorithm 1** Contrastive learning Pre-training 
**Input:** 
$H_{S}$, $H_{T}$, $H_{N}$, *P*, temperature coefficient τ, batch-size *B*, epochs *e*. 
**Output:** 
The model *M*  1:Initialization parameter *M*  2:**for** epoch in range (1,e+1) **do**  3:    **for** *i* in range (1,B+1) **do**  4:        **for** *j* in range (1,P+1) **do**  5:           # Compute the similarity between languages data  6:           $Sim\left(H_{S},H_{T}\right)=\frac{H_{S}\cdot H_{T}}{\left|\right|H_{S}\left|||\right|H_{T}\left|\right|}$  7:           # define contrastive learning loss  8:           $l_{cl}=-\log\frac{exp(Sim\left(H_{S},H_{T}\right)/\tau )}{\sum exp(Sim\left(H_{S},H_{N}\right)/\tau )}$  9:        **end for**10:    **end for**11:    # Back propagation and optimization12:    Update model M using gradient descent to minimize $l_{cl}$13:**end for**14:return M

### 3.3. Quantum Theory

This subsection focuses on how to apply quantum theory for cross-lingual ABSA, including the application of quantum projection, quantum measurement, and quantum entanglement. We first introduce the foundational concepts of quantum theory.

#### 3.3.1. The Fundamental Knowledge of Quantum Theory

Following Busemeyer et al. [35] and Fell et al. [36], we introduce key foundational concepts in quantum theory.

##### Quantum Projection

In quantum cognition, an infinite-dimensional complex vector space known as a Hilbert space H plays a pivotal role. A Hilbert space is endowed with an inner product that allows quantum states to be represented as unit vectors. Unlike classical probability, quantum probabilities derive from orthogonal basis states, with the relationship between a state vector and these bases governed by projection geometry. Notably, a single Hilbert space can be described by multiple orthogonal basis sets, which form the mathematical foundation for quantum operations such as projection and measurement. This theoretical framework is essential for understanding the application of quantum principles in our model.

##### Quantum Superposition

Quantum superposition is a core principle of quantum mechanics, suggesting that multiple states coexist within a quantum system, persisting in this state of overlap until a measurement forces a collapse into a single, well-defined outcome. A quantum system composed of an electron or a photon can remain in a superposition, where it occupies multiple possible states simultaneously. A pure state |ψ〉 is represented as a vector on the unit sphere and is defined as follows:

(3) $$|\psi \rangle =\omega_{1}|e_{1}\rangle +\omega_{2}|e_{2}\rangle +\ldots +\omega_{n}|e_{n}\rangle$$

Here, $\{|e_{1}\rangle ,|e_{2}\rangle ,\ldots ,|e_{n}\rangle \}$ represents the orthogonal basis that defines the Hilbert space, while the probability amplitudes $\left\{\omega_{1},\omega_{2},\ldots ,\omega_{n}\right\}$ are complex scalars satisfying the normalization condition $\sum_{i=1}^{n}{\left|\omega_{i}\right|}^{2}=1$, where |·| denotes the modulus of a complex number. The superposition state |ψ〉 does not coincide with any basis state $|e_{i}\rangle$. For instance, in the two-dimensional Hilbert space $H_{2}$, a pure state |ψ〉 formed by the basis states |0〉 and |1〉 can be defined as:

(4) $$|\psi \rangle =cos\frac{\theta }{2}|0\rangle +e^{i\phi }sin\frac{\theta }{2}|1\rangle$$

where $i^{2}=-1$, θ∈[0,2π] and ϕ∈[0,2π].

##### Quantum Measurement

The process of measuring a quantum system plays a crucial role in quantum theory, describing how a system initially existing in a superposition of multiple states collapses into a single, well-defined state upon observation. This process is essential for defining quantum probabilities within the framework of quantum cognition. The projection-valued measure (PVM) maps a system’s state from uncertainty to a distinct outcome by aligning it with its corresponding eigenstate. The system remains in a superposition, encompassing all possible outcomes before a measurement t is made. However, the system collapses into a particular eigenstate, when the measurement t is made.

##### Quantum Entanglement

When multiple quantum entities interact and become entangled, their individual states can no longer be fully described independently. Instead, these entities collectively form a unified quantum system represented by a pure entangled state, denoted as

(5) $$|Cl\rangle \;\in \;H_{1}⊗H_{2}⊗\cdots ⊗H_{N}$$

within the composite Hilbert space. This global pure state encapsulates non-classical correlations between subsystems, crucial for our cross-lingual ABSA framework. Importantly, while the joint entangled state |Ψ〉 remains pure, the state of any individual subsystem *i*, considered separately, is described by a mixed quantum state, namely the reduced density matrix obtained by tracing out the other subsystems:

(6) $$\rho_{i}\;=\;{\mathrm{Tr}}_{j\neq i}\left(|Cl\rangle \langle Cl|\right).$$

This mixed subsystem state reflects both local properties and the global correlations arising from entanglement. Consequently, observing or measuring one subsystem instantaneously influences the statistical description of the others, capturing precisely the deep interdependencies across languages utilized in our framework.

#### 3.3.2. Quantum Module Design

Inspired by [12], we apply quantum mechanics to solve the cross-lingual ABSA task. First, the feature representations obtained from contrastive learning pre-training serve as inputs to the quantum projection module. These features are then linearly projected into a three-dimensional Hilbert space, with measurement probabilities derived through observable operators to compute the sentiment polarity probabilities. Specifically, feature representations obtained from contrastive learning pre-training are linearly transformed into a three-dimensional Hilbert space using a projection matrix. This transformation encodes each aspect term as a quantum superposition state, enabling the model to capture multiple semantic layers simultaneously. The formal framework underlying this projection is critical for elucidating how quantum states encode linguistic features and facilitate cross-lingual knowledge transfer.

In cross-lingual aspect-based sentiment analysis, the quantum module offers two decisive benefits. Superposition naturally captures sentiment ambiguity. A three-level quantum state encodes positive, neutral, and negative polarities in a single complex vector, allowing the model to postpone a hard decision until sufficient context is observed. Classical probability vectors, by contrast, must be normalized into mutually exclusive states before inference and therefore cannot express such delayed collapse. Entanglement enables cross-lingual knowledge transfer. By preparing a global pure state, the source- and target-language subsystems are bound in a non-separable way; a measurement on the source instantly updates the target’s reduced density matrix, achieving efficient knowledge migration. Thanks to the expressive power of superposition for ambiguous sentiment and the global correlations afforded by entanglement, the quantum module delivers advantages that a purely classical probabilistic framework cannot replicate.

##### Quantum Projection Module

**Figure 3:**
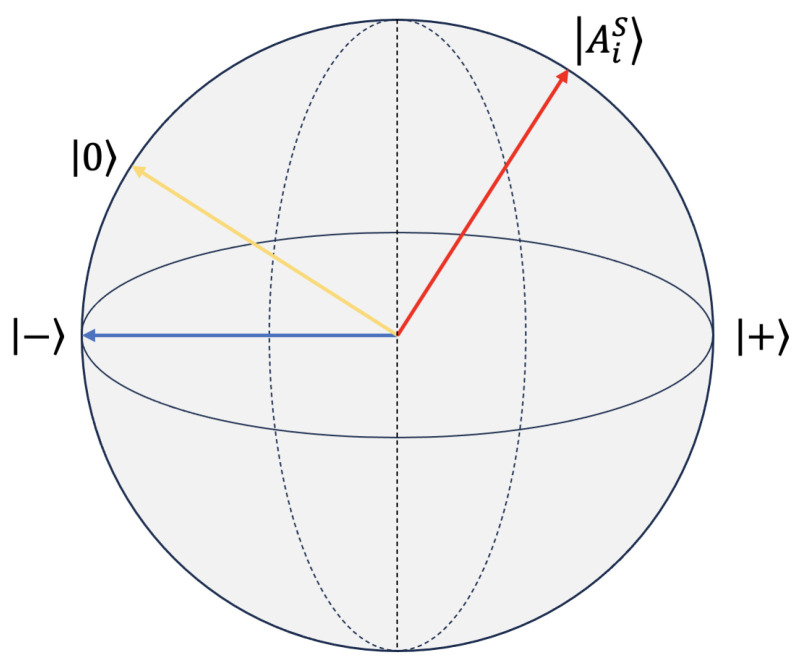
Representations of the aspect in a Hilbert space.

Each aspect word is represented as a mutually exclusive observable value within a complex-valued Hilbert space. Additionally, the feature representation *h* of words is projected into a three-dimensional Hilbert space after contrastive learning pre-training, obtaining the quantum state:

(7) $$|A_{i}^{S}\rangle =W_{p}h_{i}$$

where $W_{P}$ is the projection matrix. The Hilbert space is a three-dimensional vector space defined by the basis states {|+〉,|−〉,|−0〉}. Here, the basis states {|+〉,|−〉,|−0〉} represent positive, neutral, and negative sentiments, respectively. The aspect word $A_{i}^{S}$ is represented as a pure state within the three-dimensional Hilbert space, denoted as $|A_{i}^{S}\rangle$, Figure 3 shows the representation of aspects in Hilbert space, where each aspect word is represented by the pure state $|A_{i}^{S}\rangle$ in the three-dimensional Hilbert space with positive, neutral, and negative sentiments.

(8) $$|A_{i}^{S}\rangle =\alpha |+\rangle +\beta |0\rangle +\gamma |-\rangle$$

where ${\left|\alpha \right|}^{2}+{\left|\beta \right|}^{2}+{\left|\gamma \right|}^{2}=1$. Here, the coefficients α, β and γ indicate the contribution of each sentiment state to the overall representation.

A projection matrix $W_{p}$ is applied to the feature representation $h_{i}$ of the aspect word, yielding an unnormalized vector:

(9) $$W_{p}h_{i}=\left[\alpha_{i}^{\prime },\beta_{i}^{\prime },\gamma_{i}^{\prime }\right]$$

which is then normalized to produce the coefficients:

(10) $$\alpha_{i}=\frac{\alpha_{i}^{\prime }}{\sqrt{|\alpha_{i}^{\prime }{|}^{2}+{\left|\beta_{i}^{\prime }\right|}^{2}}+\left|\gamma_{2}^{\prime }\right|}$$

(11) $$\beta_{i}=\frac{\beta_{i}^{\prime }}{\sqrt{|\alpha_{i}^{\prime }{|}^{2}+{\left|\beta_{i}^{\prime }\right|}^{2}}+\left|\gamma_{2}^{\prime }\right|}$$

(12) $$\gamma_{i}=\frac{\gamma_{i}^{\prime }}{\sqrt{|\alpha_{i}^{\prime }{|}^{2}+{\left|\beta_{i}^{\prime }\right|}^{2}}+\left|\gamma_{2}^{\prime }\right|}$$

Defining the sentiment observable operator in the three-dimensional Hilbert space:

(13) $${\hat{S}}_{i}^{A}=\left(+1\right)|+\rangle \langle +|+\left(0\right)|0\rangle \langle 0|+\left(-1\right)|-\rangle \langle -|$$

The eigenvalues +1,0,−1 represent positive, neutral, and negative sentiments, respectively. The probability of sentiment category is calculated through quantum measurement:

(14) $$P{\left(k\right)}_{i}={\left|\langle k|A_{i}^{S}\rangle \right|}^{2}$$

where k∈{|+〉,|0〉,|−〉}, $P{\left(+\right)}_{i}={\left|\alpha_{i}\right|}^{2}$ is the probability of positive sentiment, $P{\left(0\right)}_{i}={\left|\beta_{i}\right|}^{2}$ is the probability of neutral sentiment, and $P{\left(-\right)}_{i}={\left|\gamma_{i}\right|}^{2}$ is the probability of negative sentiment. This equation quantifies the likelihood of observing each sentiment by taking the squared magnitude of the projection of the state $|A_{i}^{S}\rangle$ onto the corresponding basis state. These equations formalize how our model encodes linguistic sentiment information within a quantum framework, leveraging the principles of quantum superposition and measurement to capture complex semantic interdependencies. Consequently, the measured sentiment probability vector is defined as follows:

(15) $$p_{i}=\left[P{\left(+\right)}_{i},P{\left(0\right)}_{i},P{\left(-\right)}_{i}\right]$$

where $P{\left(+\right)}_{i},P{\left(0\right)}_{i},P{\left(-\right)}_{i}$ represent positive, neutral, and negative probability, respectively.

##### Quantum Entanglement Module

Cross-lingual ABSA tasks rely heavily on language-specific knowledge. In quantum mechanics, when multiple particles interact, each particle can acquire information about the entire quantum system through the observation of its individual state. Similarly, we create quantum entangled states between different languages to facilitate the sharing of specific linguistic knowledge. Quantum entanglement distinguishes itself from classical attention mechanisms by enabling the simultaneous representation and interaction of multilingual semantic elements within a unified quantum framework. Unlike classical attention methods, which typically aggregate information through weighted summation, quantum entanglement encodes cross-lingual semantic interactions in a high-dimensional complex vector space, allowing multiple linguistic states to coexist in superposition. This coexistence inherently accommodates linguistic ambiguity, facilitating a richer and more nuanced representation of sentiment-related information.

Therefore, Zhao et al. [12] designed a five-qubit entangled state; however, this design is fixed and assumes symmetric relationships between the languages. In reality, the relationships between languages are asymmetric. For example, the semantic overlap between English and French is more significant than that between English and Russian. Therefore, we propose an adaptive quantum entanglement module that dynamically adjusts the entanglement strength according to the similarity between languages. Additionally, we introduce quantum gates with variable parameters to ensure that the entangled state captures the specific feature across languages.

##### Language Similarity Calculation

We extract the top 100 high-frequency words $V_{i}$ for each language $L_{i}$ from the dataset and encode them as word vectors $e_{v}^{i}$. The center of language embedding can be calculated as

(16) $$E_{i}=\frac{1}{|V_{i}|}\sum_{v\in V_{i}}e_{v}^{i}$$

where $|V_{i}|=100$. The similarity between any two languages can be computed as:

(17) $$S_{ij}=\frac{E_{i}\cdot E_{j}}{\left|\right|E_{i}\left|||\right|E_{j}\left|\right|}$$

where $S_{ij}$ represents the cosine similarity between the average embeddings $E_{i}$ and $E_{j}$ of languages $L_{i}$ and $L_{j}$. The entanglement weight is formulated by

(18) $$w_{ij}=\frac{s_{ij}}{\sum_{k,l=1}^{5}s_{ik}}$$

where $w_{ij}$ is the normalized entanglement weight between languages. This normalization ensures that the weights $w_{ij}$ reflect the relative similarity of language $L_{i}$ to each target language. Since the similarity between languages varies, we define the dynamic entangled state as

(19) $$|Cl\rangle =\sum w_{ij}|\psi_{i}\rangle ⊗|\psi_{j}\rangle$$

where $|\psi_{i}\rangle =a_{i}|0\rangle +b_{i}|1\rangle$ is the quantum state of language $L_{i}$, where |0〉 represents spans the language-invariant subspace, where |1〉 represents spans the language-specific subspace, and the tensor product $|\psi_{i}\rangle ⊗|\psi_{j}\rangle$ represents the joint state of language. The weights $w_{ij}$ modulate the contribution of each language pair based on their computed similarity, allowing the entangled state to dynamically reflect the asymmetric relationships between languages. This equation defines the dynamic entangled state |Cl〉 as a weighted sum of the tensor products of quantum states from different languages. Measurement probability of $|\psi_{i}\rangle$:

(20) $$P(|\psi_{i}\rangle )=Tr_{j\neq i}(|Cl\rangle \langle Cl|)$$

where $Tr_{j\neq i}$ represents the partial trace operation, summing over all qubits except the *i*-th one (i≠j), yielding the reduced density matrix of the *i*-th subsystem. |Cl〉〈Cl| represents the density matrix of the entanglement state, a projection operator of |Cl〉. This function extracts the state of an individual language via the trace operation, enabling the computation of language-specific knowledge. Thus, language-specific knowledge representation can be defined as

(21) $$e_{i}=\langle \psi_{i}|\hat{E}|\psi_{i}\rangle$$

where $\langle \psi_{i}|$ represents the conjugate transpose (bra vector) of $|\psi_{i}\rangle$. $\hat{E}$ represents the observable operator (Hermitian operator), defined as

(22) $$\hat{E}=\sum_{k}\lambda_{k}\left|k\rangle \langle k\right|$$

where *k* represents the basis state index (k=0,1), corresponding to |0〉 and |1〉. $\lambda_{k}$ is the eigenvalue, representing the physical quantity measured in state |k〉. |k〉〈k| represents the projection operator.

**Figure 4:**
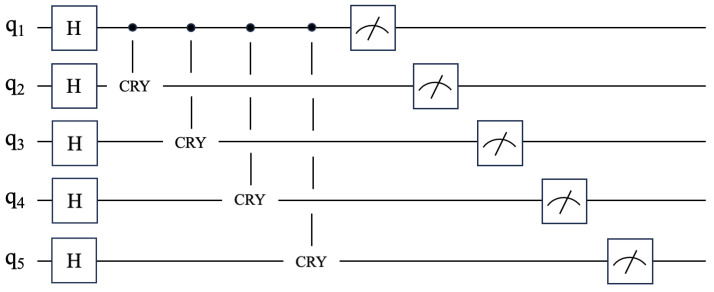
Implementation of the quantum entanglement module using a quantum circuit. H is the Hadamard gate, and CRY is the weighted revolving door.

Consequently, language knowledge vectors spanning multiple languages can be derived:

(23) $$e=[e_{1},e_{2},e_{3},e_{4},e_{5}]$$

where *e* represents a vector of knowledge representations for the five languages, each component $e_{i}$ corresponds to a language. The application circuit of entanglement state is shown in Figure 4.

By measuring its own quantum state, each particle can obtain language-specific knowledge from other particles within the system, enabling the exchange of language-specific information.

##### Fusion of Quantum Entanglement and Quantum Projection

To adapt global language knowledge to the sentiment distribution of each word, the language knowledge is mapped into the emotion space.

(24) $$e_{i}^{\prime }=W_{e}e$$

where $e_{i}^{\prime }$ represents the adjusted language knowledge vector, aligned with the sentiment space. $W_{e}$ represents the mapping matrix. Integrating the knowledge of quantum measurement and quantum entanglement:

(25) $$q_{i}=\left(1-\mu \right)p_{i}+\mu e_{i}^{\prime }$$

where $q_{i}$ represents fused sentiment probability distribution, μ represents fusion weight, μ∈[0,1], $p_{i}$ represents sentiment probability from the measure module. The normalized representation of $q_{i}$ is given by:

(26) $$q_{i}=\frac{q_{i}}{\sum_{k}q_{i,k}}$$

where $q_{i,k}$ represents the *k*-th component of $q_{i}$. To map the fused probabilities to the label space for sequence tagging, the final probability is expressed as follows:

(27) $${\hat{y}}_{i}=W_{o}q_{i}+b_{o}$$

where $W_{o}$ and $b_{o}$ represent the weight matrix and bias vector, respectively. We optimize the system using the cross-entropy function:

(28) $$L_{ce}=-\sum_{a\in D_{train}}\sum_{c\in C}y\cdot \log softmax\left({\hat{y}}_{i}\right)$$

where *a* represents aspect words, and *C* represents the number of categories. The model parameters are optimized through gradient descent by minimizing the cost function.

#### 3.3.3. Resource Estimation for the Quantum Circuit

To precisely assess the computational requirements for our proposed quantum algorithm, we provide a detailed resource estimation, including circuit width, depth, measurement strategy, and the number of shots for sufficient measurement precision. Table 1 presents the circuit width.

The circuit consists primarily of an entanglement layer that encodes cross-lingual correlations. For each token, the operation involves one single-qubit Hadamard gate to prepare superposition on the reference language qubit and four CRY gates to establish entanglement between the reference and the other four language qubits. Thus, the single-layer entanglement structure has a gate depth of approximately 5 layers.

Measurement Strategy and Shots Recommendation. In state-vector simulation, we obtain exact outcome probabilities by directly computing the squared amplitudes, so no shot-based sampling is required. On real quantum hardware, to ensure that the statistical uncertainty in the estimated probabilities 95% confidence half-width remains below 0.03, we recommend performing approximately 1024 measurement shots on all five language qubits and aggregating the observed frequencies.

## 4. Experiments

Quantum mechanics is typically represented through transformation matrices in mathematics [37]. Therefore, we represent quantum states in a linear algebraic form to evaluate the effectiveness of CLQT. A comprehensive evaluation is then performed utilizing the SemEval-2016 dataset [1] and the Amazon Reviews Corpus [38].

### 4.1. Dataset

The SemEval-2016 dataset comprises consumer opinion text in five different languages, including English, French, Spanish, Dutch, and Russian. For each language, we divide the dataset into training and test datasets. Additionally, 20% of the training samples are randomly chosen to serve as a validation dataset. Table 2 presents the SemEval-2016 dataset statistics, where “No.Sen” denotes the overall sentence count, and “No.Asp” denotes the total count of aspect terms.

The Amazon Reviews Corpus is a large-scale multilingual text classification dataset, widely used for ABSA. It includes consumer product reviews in six languages (English, German, French, Spanish, Japanese, and Chinese), annotated with star ratings ranging from 1 to 5, reflecting reviewer satisfaction. For our analysis, we categorize reviews into negative (ratings below 3), neutral (ratings equal to 3), and positive (ratings above 3). Crucially, the dataset maintains class balance, ensuring equal representation of sentiment classes. Adopting a zero-shot learning scenario, we utilize English as the source language by randomly selecting 512 reviews per sentiment category, thus compiling a total of 2560 samples for training. For validation, we randomly select 128 reviews per category from each of the remaining five languages, assembling a validation set of 3200 instances. The test set remains unchanged, comprising 5000 samples.

### 4.2. Experiment Setting

First, we translate the source language training data from the dataset into target languages to create a contrastive training dataset. The multilingual BERT and XLM-R pre-trained models are employed to encode aspects and sentences. For the contrastive learning pre-training phase, the learning rate is set to $2\times 10^{-4}$, the batch size is 16, and the temperature coefficient is 0.1. In the quantum mechanism module, the learning rate is set to $5\times 10^{-5}$, with a batch size of 16. To ensure the robustness and statistical reliability of our experimental results, we repeated each experiment five times using different random seeds. The average F1-scores, along with the corresponding standard deviations, were computed and reported. All experimental evaluations utilize the Adam optimizer.

### 4.3. Baseline Model

A comparative analysis is conducted between the introduced framework and multiple baseline approaches, along with state-of-the-art models, as follows:

- Zero-shot model [39]: This method employs annotated examples from English and utilizes them directly for predictions in another language.
- Translation-TA and Bilingual-TA [10]: These approaches leverage pseudo-labeled samples obtained via translation from a high-resource language and integrate both original and translated text for model training.
- Translation-AF and Bilingual-AF [10]: These approaches emphasize transferring labels across languages without requiring precise word-level alignment.
- ACS, ACS-DISTILL-S, and ACS-DISTILL-M [10]: These models propose a non-aligned label projection method, outperforming translation-based methods in performance. They introduce a code-switching mechanism to enrich knowledge transfer between languages using bilingual sentences.
- XLM-RoBERTa [8]: This is a transformer-based multilingual pre-trained language model that extends RoBERTa to over 100 languages, trained with a masked language modeling objective on a large-scale CommonCrawl corpus. It serves as a robust encoder for cross-lingual transfer tasks.
- CL-XABSA [20]: This model proposes two contrastive learning methods based on token labels and sentiment polarity, respectively, and integrates knowledge distillation with attention-based multilingual models to achieve cross-lingual aspect-based sentiment classification.
- CAPIT [40]: The model achieves knowledge transfer from one language to another by integrating contrastive learning with a generative prompt-based large language model.
- QPEN [12]: This quantum-enhanced network model employs quantum mechanics to solve the cross-lingual ABSA task and achieves the best performance.

### 4.4. Experimental Results

There are many non-aspect terms in sentences for cross-lingual ABSA tasks, causing a substantial imbalance in label distribution. Therefore, we use the F1 score to assess the CLQT framework. The results of the F1 score on the SemEval-2016 dataset and Amazon Reviews Corpus are shown in Table 3, Table 4 and Table 5.

Table 3 and Table 4 present F1 scores for the SemEval-2016 dataset, the proposed model achieves excellent performance across four languages, outperforming not only the baselines but also state-of-the-art models. We encode texts with different multilingual pre-trained models, including mBERT and XLM-R. We observed that CLQT outperforms both the mBERT and XLM-R models, with the improvement being more pronounced in the XLM-R model compared to mBERT. CLQT achieves better performance compared to QPEN methods, with an average absolute improvement of 1.07% in F1-score using mBERT and 2.74% using XLM-R. The results suggest that modeling aspects as quantum superposition states within a complex-valued Hilbert space, combined with facilitating the exchange of language-specific knowledge through a similarity-weighted gate quantum entanglement mechanism, can substantially improve the performance of cross-lingual ABSA tasks.

Table 5 summarizes the F1-score results of our experiments on the Amazon Reviews Corpus across five languages: German, Spanish, French, Japanese, and Chinese. Our method, denoted as CLQT, is compared against several baselines, including XLM-RoBERTa, ACS-Distill, CAPIT-base, CL-XABSA, CAPIT-large, and QPEN. Notably, CAPIT employs the mT5 [19] pre-trained encoder, which is optimally designed for generative tasks, whereas other models utilize the XLM-R pre-trained encoder.

The XLM-RoBERTa baseline achieves an average F1-score of only 50.86%, indicating that without specialized cross-lingual adaptation techniques, performance remains suboptimal. CAPIT integrates contrastive learning with large language models. In contrast, our proposed model achieves higher F1-score across all languages than CAPIT, demonstrating the benefits of combining contrastive learning with quantum networks. Similarly, the QPEN method employs the XLM-R pre-trained encoder and leverages quantum networks for performance enhancement; however, in its quantum entanglement module, different target languages are assigned the same entanglement state. By comparison, our approach significantly outperforms QPEN across all languages, yielding an average F1 improvement of 3.28%. This advantage arises not only from our dynamic entanglement module, which better adapts to real-world textual characteristics, but also from our pre-training strategy that effectively aligns semantically equivalent information across languages, thereby facilitating more effective knowledge transfer from the source to the target languages.

## 5. Analysis and Discussion

In this section, the performance of the proposed CLQT model is evaluated by verifying the effectiveness of each module, as well as ablation study, error analysis, case study, and visualization.

### 5.1. The Effectiveness of the Contrastive Learning Pre-Training

**Figure 5:**
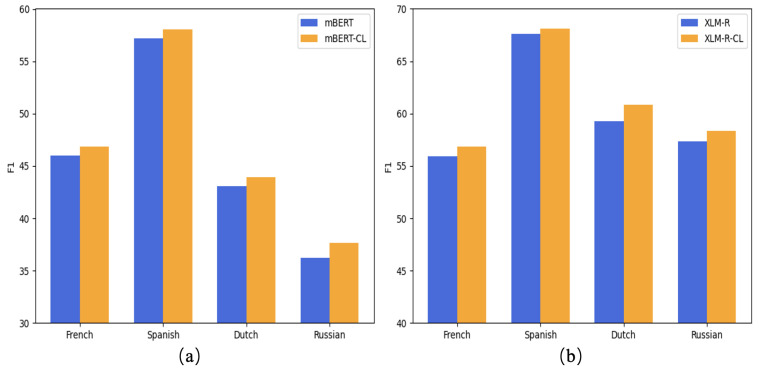
Comparison of F1 score in pre-trained language models. (**a**) presents the F1 score for mBERT and its contrastive learning-enhanced variant (mBERT-CL), while subfigure (**b**) displays the corresponding results for XLM-R and XLM-R-CL.

**Figure 6:**
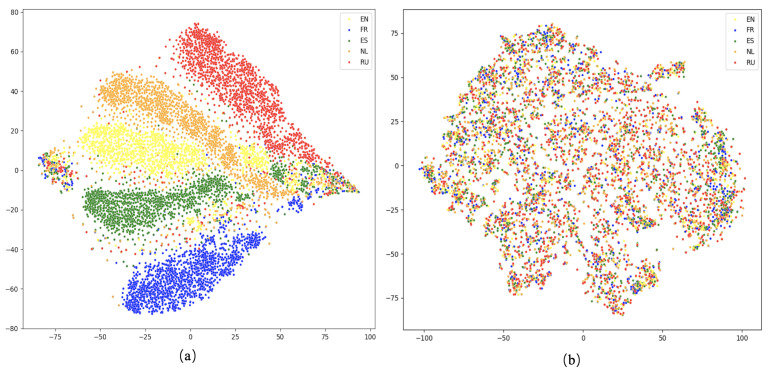
Visualization of contrastive learning pre-training. (**a**) illustrates the feature distribution in the absence of contrastive learning. (**b**) shows the feature distribution following contrastive learning-based pre-training.

To validate the effectiveness of the contrastive learning approach in cross-lingual aspect-based sentiment analysis, we conduct both F1-score evaluations and visualization experiments on the SemEval-2016 dataset. The F1-score results are presented in Figure 5, while the visualization results are shown in Figure 6.

In Figure 5, blue bars represent the baseline pre-trained models, while orange bars indicate the models augmented with contrastive learning (mBERT-CL and XLM-R-CL). The x-axis denotes the four evaluated languages, French, Spanish, Dutch, and Russian, while the y-axis reflects F1 scores for the cross-lingual aspect-based sentiment classification task.

Across all languages, models enhanced with contrastive learning consistently outperform their baseline counterparts. For instance, in Spanish, mBERT-CL achieves an F1 score of nearly 58%, compared to approximately 56% for the original mBERT. A similar trend is observed with XLM-R, particularly in lower-resource languages such as Russian and Dutch, where contrastive learning leads to notable improvements. Even in Spanish, the best-performing language overall, contrastive learning yields consistent gains.

These F1 score results strongly validate the effectiveness of contrastive learning pre-training, demonstrating its ability to effectively reduce cross-lingual semantic gaps and significantly enhance the generalization capabilities of pre-trained language models across diverse linguistic settings.

Figure 6 presents the t-SNE visualizations of multilingual feature representations. These visualizations are generated based on the training dataset, which includes English (EN) samples and their corresponding translations in French (FR), Spanish (ES), Dutch (NL), and Russian (RU), encoded using the pre-trained XLM-R model.

(a) displays the feature distribution without the application of contrastive learning. Although the sentences across different languages are semantically equivalent, their encoded representations exhibit clear language-specific clustering, where samples from each language form separate clusters. This language-dependent distribution suggests that, without contrastive learning, the model struggles to align cross-lingual semantics effectively, potentially introducing noise during knowledge transfer and negatively impacting cross-lingual aspect-based sentiment classification. In contrast, (b) presents the feature distribution after applying contrastive pre-training. Representations of semantically equivalent samples across different languages are noticeably closer in the feature space, forming well-aligned, cross-lingual clusters. This indicates that contrastive learning facilitates the model in capturing language-invariant semantic information, thereby enhancing cross-lingual alignment.

These findings corroborate the quantitative improvements observed in Figure 5, confirming the effectiveness of contrastive learning in improving cross-lingual semantic consistency. By reducing semantic gaps between translated instances, contrastive learning helps mitigate translation-induced noise and enhances the generalization of the model and accuracy in multilingual aspect-based sentiment classification.

### 5.2. The Effectiveness of Quantum Mechanism Dynamic Entanglement

**Figure 7:**
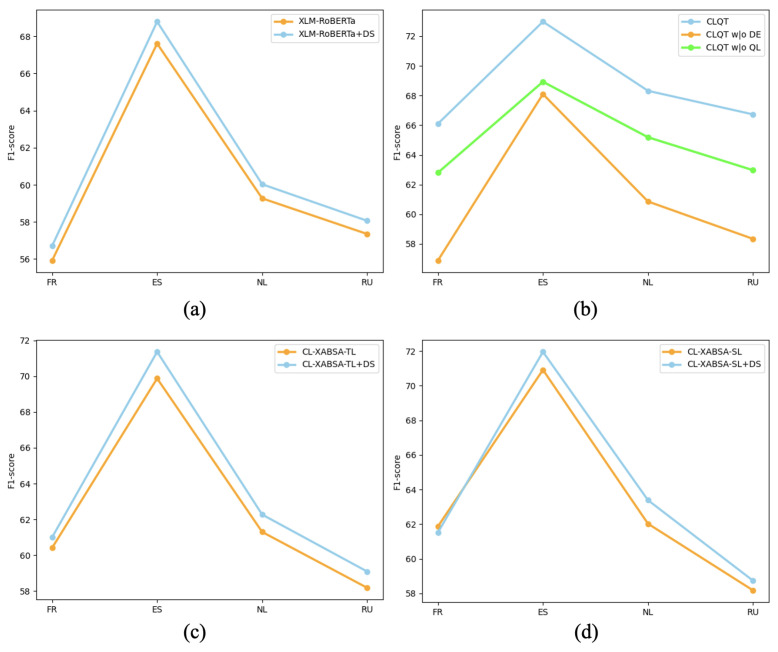
Performance comparison of different models with the addition of the dynamic similarity module. (**a**) XLM-RoBERTa and its derivative models; (**b**) CLQT together with its companion approaches; (**c**) CL-XABSA-TL and its associated variants; and (**d**) CL-XABSA-SL along with its related methods.

To assess the effectiveness of the dynamic entanglement mechanism proposed in our quantum framework, we devise a series of experiments on the SemEval-2016 dataset. These experiments examine the impact of integrating the dynamic similarity weighting strategy across various model architectures. The corresponding F1 scores are reported in Figure 7, providing empirical evidence of the utility of this component in enhancing cross-lingual aspect-based sentiment classification performance.

Subgraph (a) presents the experimental results of integrating the widely used multilingual model XLM-RoBERTa with the proposed Dynamic Similarity Weights (DS) module. XLM-RoBERTa captures contextual semantic representations via a multilingual attention mechanism. When enhanced with the DS module, it dynamically adjusts the fusion weights between source and target language representations based on their similarity, resulting in improved F1 performance across various languages. However, since the fusion is still performed via linear weighting, the overall performance gain remains relatively limited.

Subgraphs (c) and (d) further explore the impact of the DS module on two attention-based baseline models, CL-XABSA-TL and CL-XABSA-SL. In both cases, the inclusion of DS leads to measurable performance gains in French, Spanish, Dutch, and Russian, though the improvements are constrained by the limited capacity of attention mechanisms to capture complex semantic dependencies.

Subgraph (b) illustrates the performance of the proposed CLQT model and its ablated variants. Removing the quantum module entirely (CLQT w/o QM) results in the most pronounced performance degradation, underscoring the pivotal role of quantum components in capturing semantic relationships. A partial ablation, excluding only the dynamic entanglement module (CLQT w/o DE), still yields competitive results, exceeding those of XLM-R and CL-XABSA, but falls short of the full CLQT configuration. These findings highlight the unique contribution of dynamic quantum entanglement in modeling semantic alignment across languages. The complete CLQT model achieves the highest F1 scores across all languages, confirming the synergistic benefit of combining quantum projections, entanglement, and adaptive similarity weighting.

While conventional approaches such as attention or linear weighting can integrate cross-lingual features, they often rely on local information and struggle to fully model nonlinear semantic mappings across languages. In contrast, the proposed dynamic quantum entanglement mechanism constructs global entangled states in Hilbert space guided by dynamic similarity, enabling multilingual features to reside in a unified, cooperative quantum representation. This facilitates a more precise modeling of deep semantic dependencies during knowledge transfer. Experimental results confirm that the quantum entanglement mechanism, driven by dynamic similarity, significantly enhances the expressive power and generalization performance of the model in cross-lingual aspect-based sentiment classification tasks.

### 5.3. Ablation Study

To further explore the contribution of each module in the model, we designed several variants. The experimental results for each variant are presented in Table 6 and Table 7. Specifically, Variant 1, only containing the multilingual pre-training module, exhibits the poorest performance, whereas the inclusion of additional modules generally results in improved performance. Variant 2, which incorporates a contrastive learning pre-training module, demonstrates a significant improvement in experimental performance compared to Variant 1. This indicates that the proposed contrastive learning pre-training model effectively reduces the semantic distance between languages through knowledge transfer and thereby improves the performance of the model. Variant 3 removes the contrastive learning pre training and dynamic entanglement modules from the proposed CLQT model. Variant 4 adds a contrastive learning pre-training module on the basis of Variant 3. The results of variant 3 indicate that our proposed quantum mechanism module successfully integrates syntactic and semantic information from five languages. Comparing variant 4 with the CLQT model, it was found that applying the similarity rotation gate to quantum dynamic entanglement is effective and improves the performance of the model, which could better recognize aspect words and emotions.

### 5.4. Case Study

Table 8 presents three examples from the QPEN and our proposed CLQT models. −1, 0, and 1 represent negative, neutral, and positive sentiment, respectively. The results in brackets are aspect terms and sentiment polarities. From the first two examples, it is evident that both the QPEN and CLQT models effectively identify aspect terms in target language sentences, even when multiple aspect terms are present. Example 3 is a Dutch sentence that contains two aspects wijnkaart and wijnen per glass. It is observed that our proposed CLQT model successfully identifies both aspect terms and sentiment polarities, whereas the QPEN model only identifies the wine list aspect term. The enhanced performance of the CLQT model can be attributed to two primary factors. First, dynamic entanglement captures the relationships between languages more effectively than static entanglement states. Second, the contrastive learning pre-training module substantially improves the transfer of knowledge from the source language to the target language.

### 5.5. Error Analysis

**Figure 8:**
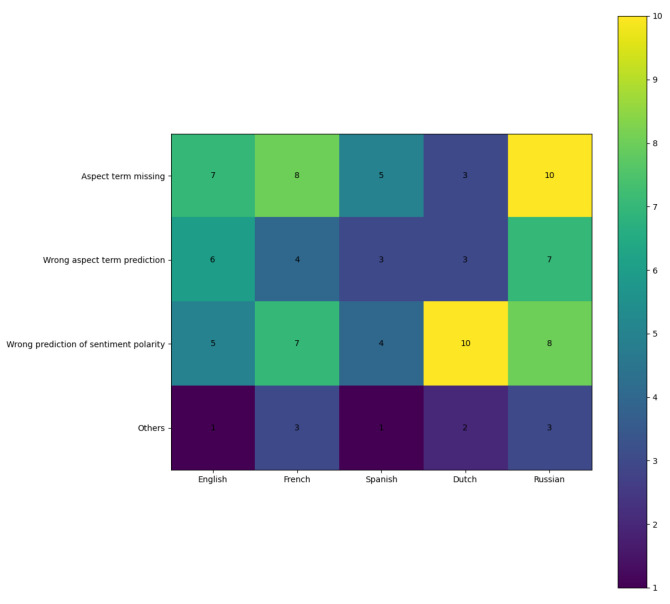
Error type distribution heatmap.

An error analysis of CLQT performance is conducted by selecting 20 failed instances from each language, resulting in a total of 100 samples. The distribution of different error types is depicted in Figure 8. A manual review indicates that the majority of errors fell into the following categories:

- Aspect item missing: Such errors are common and may arise from variations in language distribution, particularly due to structural inconsistencies across languages. It leads to a loss of crucial knowledge during the transfer from the source language to the target language, which impacts performance.
- Wrong aspect item prediction: Incorrect aspect word predictions indicate a deviation in cross-lingual knowledge transfer, and the use of quantum projection and superposition introduces noise that further compromises the accuracy of this process.
- Wrong prediction of emotional polarity: Incorrect predictions of emotional polarity are frequently observed, particularly in texts that are challenging to interpret, such as those containing satirical expressions.
- Other errors

According to error analysis for our model, we plan to pursue several research directions to further mitigate error propagation and enhance the robustness of our framework. We will refine our feature extraction techniques by incorporating more advanced syntactic and semantic representations that capture subtle linguistic cues more effectively. This may involve leveraging state-of-the-art contextual embeddings models to reduce the incidence of missing aspect items. We will develop advanced disambiguation strategies to enhance sentiment polarity prediction, particularly in challenging cases such as texts with ambiguous or satirical expressions. These strategies may combine rule-based approaches with quantum techniques and integrate external knowledge sources to provide richer contextual cues.

These enhancements are expected to significantly reduce error and further improve the overall performance and robustness of our cross-lingual ABSA framework.

### 5.6. Visualization

**Figure 9:**
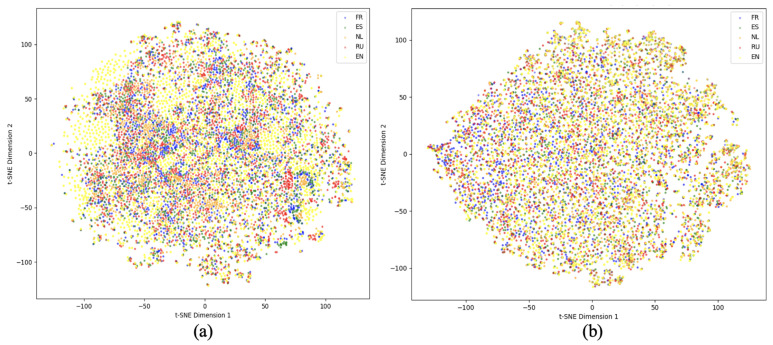
We visualize the test set by applying t-SNE to the sentence embeddings generated by CLQT. In this visualization, panel (**a**) illustrates the embedding plot produced by the XLM-R model, while panel (**b**) depicts the plot obtained from CLQT.

To systematically analyze the behavior of our framework, we utilize t-SNE to visualize the feature representations, as presented in Figure 9. Panel (a) shows the embedding distribution from the XLM-R model, while panel (b) depicts the visualization produced by CLQT. Compared with the baseline XLM-R, CLQT yields a more uniform distribution representation between across languages. This indicates that the proposed contrastive learning pre-training and quantum modules facilitate the sharing of language-specific knowledge, ultimately leading to more accurate predictions for the cross-lingual ABSA task.

Specifically, the t-SNE visualizations provide a qualitative insight into the embedding space and demonstrate the effectiveness of our cross-lingual alignment strategy. In our experiments, we compare the t-SNE plots of token embeddings generated by a baseline XLM-R with those produced by our proposed CLQT model. The baseline embeddings exhibit distinct clusters corresponding to individual languages, reflecting the persistence of language-specific features and limited cross-lingual transfer. In contrast, the embeddings derived from CLQT show a much more uniform distribution across languages, with clusters that are more intermixed and aligned. Moreover, the visualization highlights that the utilization of contrastive learning pre-training and dynamic quantum entanglement in the model contributes to a more uniform distribution of representations across languages. This uniformity indicates that the framework successfully bridges the semantic gap between source and target languages, ensuring that similar sentiment expressions are aligned regardless of language.

Overall, the t-SNE visualizations reinforce the quantitative improvements reported in our experimental results by offering a visual confirmation that our integrated contrastive learning and quantum mechanism approach effectively bridges the gap between languages.

## 6. Conclusions

In this study, we introduced CLQT, an innovative framework designed to enhance cross-lingual ABSA through the integration of contrastive learning pre-training and quantum mechanisms. Specifically, our framework leverages contrastive learning to align representations of source language data and its translations effectively, thereby facilitating more robust semantic knowledge transfer across languages. Additionally, we incorporated quantum projection and dynamic quantum entanglement modules, enabling the model to automatically adjust entanglement strength based on linguistic similarities. This approach allows for more precise encoding of linguistic interactions and nuanced sentiment dependencies that classical methods may overlook. Our extensive experiments conducted on two benchmark datasets, SemEval-2016 and Amazon Reviews Corpus, demonstrate the effectiveness of CLQT. The model consistently outperformed existing state-of-the-art methods, showcasing marked improvements in F1 scores across multiple languages. Ablation studies further highlighted the critical role of the dynamic quantum entanglement module, affirming its significant contribution to performance enhancement.

However, our analysis also revealed several limitations. Errors in aspect extraction and sentiment classification often arise from structural and semantic divergences across languages, as well as from the inherent ambiguities of natural text. To address these challenges, we will refine our feature-extraction pipeline by incorporating advanced syntactic and semantic representations, such as graph-based linguistic structures and deep contextual embeddings, and by developing translation-noise mitigation strategies, including iterative back-translation and adversarial data augmentation. We also plan to explore deeper, error-mitigated entanglement circuits and to extend CLQT into a fully quantum, end-to-end sequence-labeling framework that jointly encodes aspect extraction and sentiment prediction. Finally, we will investigate the broader applicability of quantum-inspired representations across other cross-lingual NLP tasks, aiming to harness their power for modeling complex linguistic phenomena.

## Figures and Tables

**Table 1:** Circuit width.

Sub-Register	Logical Objects	Physical Encoding	Qubit Count
Language register	L=5 languages	1 qubit per language	5
Sentiment register	3-level (+,0,−)	mapped post-entanglement	2
controlled rotations and read-out	transient	reused and reset	≤1

These two qubits are not part of the core entanglement network but are used later for sentiment measurement.

**Table 2:** SemEval-2016 consumer reviews dataset.

		English	French	Spanish	Dutch	Russian
Train	No.Sen	2000	1664	2070	1722	3655
	No.Asp	1743	1641	1856	1231	3077
Test	No.Sen	676	668	881	575	1209
	No.Asp	612	650	713	373	949

**Table 3:** CLQT uses mBERT pre-trained model in SemEval-2016 experimental results.

Methods	mBert				
	French	Spanish	Dutch	Russian	Avgerage
Zero-shot	45.60	57.32	42.68	36.01	45.40
Translation-TA	40.76	50.74	47.13	41.67	45.08
Bilingual-TA	41.00	51.23	49.72	43.67	46.41
Translation-AF	48.03	59.74	49.73	50.17	51.92
Binglingual-AF	48.05	60.23	49.83	51.24	52.34
ACS	49.65	59.99	51.19	52.09	53.23
ACS-Distill-S	52.23	62.04	52.72	53.00	55.00
ACS-Distill-M	52.25	62.91	53.40	54.58	55.79
CL-XABSA-TL	48.53	60.64	50.96	50.77	52.73
CL-XABSA-SL	49.50	61.62	50.64	50.65	53.10
QPEN	53.27	63.84	54.61	55.36	56.77
CLQT (ours)	**54.34**	**65.01**	**55.73**	**56.27**	**57.84**

**Note:** Values in bold represent the optimal results.

**Table 4:** CLQT uses XLM-R pre-trained model in SemEval-2016 experimental results.

Methods	XLM-R				
	French	Spanish	Dutch	Russian	Avgerage
Zero-shot	56.43	67.10	59.03	56.80	59.84
Translation-TA	47.00	58.10	56.19	50.34	52.91
Bilingual-TA	49.34	61.87	58.64	52.89	55.69
Translation-AF	57.07	66.61	61.26	59.55	61.12
Binglingual-AF	57.91	68.04	60.80	60.81	61.89
ACS	59.39	67.32	62.83	60.97	62.63
ACS-Distill-S	61.00	68.93	62.89	60.97	63.45
ACS-Distill-M	59.90	69.24	63.74	62.02	63.73
CL-XABSA-TL	60.41	69.87	61.30	58.82	62.60
CL-XABSA-SL	61.87	70.95	62.03	58.18	63.26
QPEN	63.21	71.59	66.16	64.52	65.79
CLQT (ours)	**66.10**	**72.98**	**68.32**	**66.73**	**68.53**

**Note:** Values in bold represent the optimal results.

**Table 5:** The results of experiment on Amazon Reviews Corpus dataset.

Methods	German	Spanish	French	Japanese	Chinese	Avgerage
CAPIT-base	76.99	75.64	75.36	73.48	68.78	74.09
CAPIT-large	78.34	75.56	76.90	73.54	70.60	75.00
XLM-RoBERTa	55.52	51.96	52.42	51.30	48.86	50.86
ACS-Distill-S	68.71	67.29	64.02	61.85	58.67	64.05
ACS-Distill-M	70.58	68.97	66.35	63.14	60.72	65.95
CL-XABSA-TL	68.32	67.70	63.47	61.16	57.89	63.71
CL-XABSA-SL	69.59	69.04	64.70	62.63	58.07	64.80
QPEN	78.98	78.84	75.93	72.19	68.20	74.83
CLQT	**82.02**	**81.85**	**79.19**	**75.77**	**71.93**	**78.15**

**Note:** Values in bold represent the optimal results.

**Table 6:** The results of ablation study on mBERT pre-training model.

Methods	mBert				
	French	Spanish	Dutch	Russian	Average
Variant1	46.01 ±2.23	57.19 ±1.67	43.05 ±2.44	36.24 ±1.89	45.62 ±2.06
Variant2	46.82 ±1.94	59.71 ±1.42	43.94 ±2.17	37.65 ±1.63	47.03 ±1.79
Variant3	53.27 ±1.88	63.84 ±1.39	54.61 ±2.12	55.36 ±1.58	56.97 ±1.74
Variant4	53.80 ±1.49	64.27 ±1.08	55.14 ±1.66	55.92 ±1.27	57.28 ±1.38
CLQT (ours)	54.34 ± 0.95	65.01 ± 1.30	55.73 ± 1.35	56.27 ± 0.52	57.84 ± 1.03

**Table 7:** The results of ablation study on XLM-R pre-training model.

Methods	XLM-R				
	French	Spanish	Dutch	Russian	Average
Variant1	55.92 ±2.16	67.61 ±1.33	59.27 ±2.23	57.35 ±1.87	60.04 ±1.90
Variant2	56.88 ±1.82	68.10 ±1.09	60.86 ±1.94	58.34 ±1.61	61.05 ±1.62
Variant3	63.21 ±1.78	71.59 ±1.06	66.16 ±1.91	64.52 ±1.58	66.37 ±1.58
Variant4	64.36 ±1.47	71.93 ±0.88	67.25 ±1.62	65.49 ±1.36	67.26 ±1.33
CLQT (ours)	66.10 ± 1.47	72.98 ± 1.14	68.32 ± 1.84	66.73 ± 1.23	68.53 ± 1.42

**Table 8:** Case study.

Languages	Sentences	QPEN	CLQT
SP	La comida estuvo muy sabrosa.	(comida 1)	(comida 1)
FR	Le cadre et le personnel sont agréables.	(cadre 1, personnel, 1)	(cadre 1, personnel, 1)
DU	Geen kennis van de wijnkaart laat staan van de wijnen per glass.	(wijnkaart −1)	(wijnkaart −1, wijnen per glass −1)

## Data Availability

All original contributions of this study are incorporated in this article; any further inquiries may be directed to the corresponding author.
